# Supplementary material for: The Antibacterial and Wound Healing Properties of Natural Products: A Review on Plant Species with Therapeutic Potential against Staphylococcus aureus Wound Infections
Source: Plants (Basel). 2023 May 29;12(11):2147. doi: 10.3390/plants12112147 (PMC10255540; doi:10.3390/plants12112147)
Supplement: Supplementary file 1 [file plants-12-02147-s001.zip › plants-2374141-supplementary.pdf]

Table S1: Antibacterial and healing properties of plant species against *Staphylococcus aureus* wound infections, covering studies from January 2015 to March 2023: extracts and main phytochemicals, *in vitro* and *in vivo* analyses

Species (Family)	Plant part/ extracts	Phytochemicals	*In vitro* assays	*In vivo* wound model: doses, treatment protocols and findings	Ref.
Allium stipitatum (Amaryllidaceae)	Fresh bulbs<br><br>Hexane (H) and dichloromethane (DM) extracts	Essential fatty acids, decanoic acid, 9,12-octadecadienoic acid, monoterpenoids, saturated/unsaturated fatty acids, organosulfur compounds, aromatic alcohol, synthetic intermediates, organic ester fatty acids, and aromatic hydrocarbons were identified. High concentrations of γ-hexalactone (19.37%), 9,12-octadecanoic acid (19.17%) and hexadecanoic acid (19.37%).	H and DM MIC values of 32.0 and 64.0 µg/mL, respectively, for MRSA ATCC 43300. MBC = 128.0 µg/mL for both extracts. CC_{50} values of H and DM for VERO cells were 383.4 and 390.6 µg/mL, respectively.	Burn wound in BALB/c female mouse MRSA ATCC 43300 inoculation was carried out 30 min after injury. The topical treatment (twice a day/20 days) with 20 mg of the ointments (containing 0.5, 1.0 or 2.0% of H or DM extracts) started 24 h post-infection.	[48]
Aloe spp.<br>*A. tormentorii* (Marais) L.E.Newton & G.D.Rowley<br>*A. purpurea* Lam.<br>*A. macra* Haw.<br>*A. lomatophylloides Balf.f*<br>*A. vera*<br>(Asphodelaceae)	Leaves<br><br>Methanolic extract	Anthraquinones, anthrones, chromones and flavone C-glycosides were detected.	MIC values ranged from 1.56 mg/mL (*A. purpurea*) to 25.0 mg/mL (*A. vera*) for *S. aureus* ATCC 12600. Methanolic extracts of *A. purpurea* and *A. vera* displayed wound healing activityt in HaCaT scratched monolayers. Except for *A. purpurea*, all methanolic extracts were *non*-toxic against HL60 and MRC 5 cells.	ND	[49]
Althaea officinalis L. (Malvaceae)	Leaves<br><br>Hydroethanolic extract	NI	MIC and MBC values of hydroethanolic extract for *S. aureus* ATCC 25923 were 330.0 ± 0.1 µg/mL and 660.0 ± 0.2 µg/mL, respectively.	Excision wound in *Wistar* male rat 0.5 g of the plant extract every two days. The ointment containing zinc-oxide was used as reference.	[50]

Anethum graveolens L (*Apiaceae* family)	NI<br><br>Essential oil	15 phytochemicals accounted for 99.5% of the total essential oil. Monoterpene hydrocarbons: α-phellandrene (47.3%), *p*-cymene (18.5%) and limonene (5.7%). Monoterpenes: carvone (14.1%) and dill ether (7.5%).	ND	Excision wound in male BALB/c male mouse<br>MRSA isolated from burn wound inoculation was carried out 5 min after injury. The topical treatment with ointment containing 2.0 or 4.0% of essential oil. 2.0% mupirocin® ointment was used as reference drug.	[51]
Angelica dahurica Bentham et Hooker (Umbelliferae)<br>*Rheum officinale* Baill. (Polygonaceae)	NI<br><br>Hydroethanolic extract	Rhein (95 μg/mL), aloe-emodin (32 μg/mL), chrysophanol (12 μg/mL), of emodin (36 μg/mL) of physcion (3 μg/mL).	The mixture inhibited the growth of *S. aureus* ATCC 29213 by disk-diffusion method.	Excision wound in Sprague Dawley male rat<br>Treatment (once a day/7 days) with the mixture started 24 h after wound infection with *S. aureus* ATCC 29213.	[52]
Balanites aegyptiaca (Balanitaceae)	Bark<br><br>Hydroethanolic extract	NI	MIC and MBC values of the extract ranged from 2.5 to 12.5 μg/mL and 2.5 to 50.0 μg/mL, respectively, against<br>45 *S. aureus* (including MRSA).	ND	[54]
Bergia ammannioides Henye ex Roth. (Elatinaceae)	NI<br><br>Ethanolic extract (E):<br>*N*-hexane fraction (H); and ethyl-acetate fraction (EA)	β-sitosterol, lupeol, cyclolaudenol, and cycloartenol were isolated from *n*-hexane fraction of the ethanolic extract. Quercetin, ellagic acid, kaempferol-3-O-α-L-rhamnoside, and quercetin-3-O-α-L-rhamnoside were isolated from ethyl-acetate fraction of the ethanolic extract.	MIC values E, H and EAd for *S. aureus* ATCC 4175 were 178.0, 104.0, 152.0 μg/mL, respectively.<br>The extracts presented antioxidant activity.	Excision wound in Sprague-Dawley male rat and *Swiss* albino mouse<br>Ointment (containing 5.0% or 10.0% of E or H or EA fractions) application was carried out once a day/10 days.<br>A dose dependent healing activity was observed for all ointments.	[56]
Bursera morelensis (Burseraceae)	Barks<br><br>Methanolic extract	Total phenolic and flavonoid compounds were determined. Catechin was identified.	*In vitro* assays<br>MIC values of 4.0 mg/mL, 1.0 mg/mL and 2.0 mg/mL were determined for *S. aureus* ATCC	Incisional wound in CD-1 male mouse<br>0.5 mL carbomer-based gel loaded with 10.0% of plant extract were	[57]

			25923, one MSSA clinical isolate, and one MRSA clinical isolate, respectively.	applied twice a day/10 days. Recoveran was used as reference drug.	
Calophyllum inophyllum (Calophyllaceae)	Seeds<br><br>Oil obtained from five geographic regions	Resinous fraction	MIC values of the oils ranged from 0.01% to 0.1% for *S. aureus* ATCC 9144 oxford, *S. aureus* ATCC 25923 and five clinical isolates.<br>All oils were non-toxic to keratinocyte cell; accelerated wound healing in HaCaT scratched monolayers; and increased the β-defensin release by U937 derivative macrophage cells.	ND	[58]
Capsicum annuum L. (Solanaceae)	Dried fruits<br><br>Methanolic extract	NI	MIC and MBC values ranged from 64.0 to 256.0 μg/mL and 256.0 to 1024.0 μg/mL, respectively, for *S. aureus* ATCC 25923 and two clinical isolates.<br>At MIC value, the extract caused around 50.0% reduction of biofilm viability.<br>Reduction of the bacterial ATPases/H^+ proton pump and dehydrogenase activities;<br>Induction of proteins and reducing sugar leakage in bacteria.	Excision wound model in *Wistar* rat Carbopol-based gel (containing 1.0%, 5.0% or 10.0% of methanolic extract) application was carried out once a day/20 days 24 h post-infection with *S. aureus*. Clindamycin (Aclin gel) was used as reference drug.	[59]
Carthamus tinctorius L. (Asteraceae)	Seeds<br><br>Oil	Total phenolic, flavonoid, carotenoid and chlorophyll contents were determined.	*In vitro* assays<br>The oil presented antioxidant activity, but did not inhibit the growth of *S. aureus.*	ND	[60]

Chamaecyparis obtuse (Cupressaceae)	NI<br><br>Lipid extract: 10 major lipid components	38 phytochemicals were identified, and the 10 major lipids in descending order of concentration were: terpinyl acetate, guaiol, elemol, sabinense, palmitic acid thujopsene, totarol, 9-octadecenamide, β-pinene, and cembrene.	0.01% and 0.02% lipid extracts presented bactericidal activity against *S. aureus* ATCC BAA-29213 and MRSA ATCC BAA-1556 strains after one/two hours-incubation. The lipid mixture was non-toxic to human primary keratinocytes; accelerated keratinocytes migration in scratched monolayers; inhibited keratinocytes death induced by *S. aureus* α-toxin.	Excision wound model in hairless female mouse (Crl: SKH1-*Hrhr*)<br>0.02% solution of the 10-lipid mixture in buffered saline were applied on wounds non-infected or infected with MRSA (ATCC BAA-1556) three times a day/10 days.	[61]
Commiphora gileadensis (Burseraceae)	Leaves and branches<br><br>Methanolic extract	Ceramide (69.0%)<br>Hexosylceramide (18.0%)<br>Phosphatidylethanolamine (7.6%)<br>Other (5.4%)	ND	Excision wound model in BALB/c mouse<br>Treatment (once a day) with methanolic extract (4 mg/g of the animal) started 6 h after *S. aureus* inoculation. The results were comparable with gentamicin (reference drug) treatment. Anti-inflammatory and antibacterial activities contributed for wound healing	[62]
Cratylia mollis (Fabaceae)	Seeds<br><br>Lectin Cramoll	Lectin	ND	Infection model in *Tenebrio monitor* larvae<br>Treatment with lectin (125.0, 250.0 or 500.0 μg/kg of the larvae) started 2 h after *S. aureus* inoculation. A significant difference was observed in the survival of larvae infected with *S. aureus* and treated with 500.0 μg/kg of lectin, when compared with the infected and untreated group.	[63]

				Excision wound model in *Swiss* mouse<br>Treatment (once a day/10 days) with lectin (5.0 µg/animal) started 24 h after *S. aureus* inoculation. The treatment reduced both the levels of TNF-α, NO and VEGF, and the *S. aureus* load at the wound site.	
Cyrtocarpa procera Kunth (Anacardiaceae)	Barks<br><br>Methanolic extract	Total phenolic and flavonoid compounds were determined. Catechin was identified.	MIC = 0.5 mg/mL for *S. aureus* ATCC 25923 and one MSSA clinical isolate. MIC = 1.0 mg/mL for one MRSA clinical isolate.<br>The extract presented antioxidant activity.	Incisional wound in CD-1 male mouse<br>0.5 mL carbomer-based gel loaded with 10.0% of plant extract were applied twice a day/10 days.<br>Recoveran was used as reference drug.	[57]
Elaeis guineensis Jacq. (Arecaceae)	Leaves<br><br>Methanolic extract	NI	ND	Excision wound model in Sprague Dawly male rat<br>Ointment (containing 10.0% of methanolic extract) application was carried out once a day/20 days and started 24 h after *S. aureus* infection.<br>Commercial antiseptic ointment containing Povidone-Iodine (10% w/v) (BETADINE® was used as reference drug.	[64]
Entada phaseoloides (L.) Merr. (Leguminosae)	NI<br><br>Hydroethanolic extract	Tannin (76.18%)	MBC = 1.5 mg/mL for *S. aureus* ATCC 25923.<br>Cell proliferation and collagen deposition in NIH3T3 mouse fibroblast scratched monolayer.	Excision wound model in SD male rat<br>Ointment (containing 0.5 mg of plant extract) was applied once a day after *S. aureus* infection<br>Bactroban ointment was used as reference.	[65]

Garcinia mangostana Linn (Clusiaceae)	Fruit pericarp<br><br>Ethanolic extract	NI	MIC and MBC values of the plant extract ranged from 0.02 to 1.25 mg/mL and 0.03 to 5.0 mg/mL, respectively, for *S. aureus* ATCC 9144 and *S. aureus* ATCC 23235, and MRSA clinical isolates.	Tap stripping wound model in ICR male mouse<br>Topical formulations (100 μL) were prepared in 10.0% ethanol in propylene glycol (vehicle) containing 10.0% ethanolic extract or 1.32% α-mangostin (major xanthone in the fruit pericarp) and applied on wounds 24 h after of MRSA DMST 20651 once a day/10 days.	[66]
Hypericum perforatum (Hypericaceae)	Aerial parts<br><br>Aqueous extract	NI	The aqueous extracts (7.5, 15.0, 30.0, 60.0, 100.0 and 150.0 mg/mL) inhibited the growth of *S. aureus* ATCC 29213 in a concentration-dependent manner (evaluated by the well-diffusion assay).	ND	[68]
Jatropha multifida L. (Euphorbiaceae)	Leaves<br><br>Ethanolic extract	NI	MIC and MBC values ranged from 2.5 to 12.5 μg/mL and 3.12 to 12.5 μg/mL, respectively for 45 *S. aureus* (including MRSA) isolates.<br>The extract exhibited anti-inflammatory activity in a carrageenan and histamine-induced paw edema method in a rat model.	ND	[69]
Jatropha neopauciflora L. (Euphorbiaceae)	NI<br><br>Latex	Total phenolic and flavonoid compounds, and carbohydrates and proteins were determined.<br>Catechin and catechol were identified.	MIC and MBC values of 2.0 and 4.0 μg/mL, respectively were determined for *S. aureus* ATCC 29213 and one clinical isolate.<br>Latex presented antioxidant activity.	Wound model in CD1 *Mus musculus* mouse<br>Topical treatment with 50%, 75% or 100% of latex was applied every 12 h for 10 days and induced complete wound healing. Recoveran (reference	[70]

				drug) caused 85.0% of wound contraction.<br>Oral administration of 500.0 or 750.0 mg/kg of animal caused around 65% and 70% inflammation reduction in a carrageenan-induced edema model, whereas topical administration caused around 25% inflammation reduction.	
Lawsonia inermis<br>Henna (Lythraceae)<br>*Azadirachta indica*<br>Neem (Meliaceae)<br>*Achyranthus aspera*<br>Telenge (Amaranthaceae)	Leaves<br><br>Methanolic extracts	Alkaloids, terpenoids, phenols, tannins and steroids were qualitatively identified	Weak antibacterial activity of all plant extracts was observed by using the agar well diffusion assay, compared to the cefoxitin. The MIC values of 6.25, 25.0 and 50.0 mg/mL were identified for *L. inermis*, *A. indica* and *A. aspera*, respectively, for *S. aureus.*	ND	[46]
Matricaria chamomilla<br>(Asteraceae)	Whole Flowers<br><br>Aqueous extract	NI	The aqueous extracts (7.5, 15.0, 30.0, 60.0, 100.0 and 150.0 mg/mL) did not inhibit the growth of *S. aureus* ATCC 29213 (evaluated by well-diffusion assay).	ND	[68]
Moringa oleifera<br>(Moringaceae)	Leaves<br><br>Aqueous extract	NI	ND	Excision wound in *Wistar* rat<br>Oral treatment (twice a day/7 days) with the aqueous extract (150 mg/kg of animal) started 48 h after *S. aureus* inoculation did not presente healing activity.	[71]
	Leaves	Alkaloids, flavonoids, polyphenols, tannins and steroids were qualitatively identified	MIC and MBC values of the methanolic extract for *S. aureus*	Excision wound model in *non-*diabetic and diabetic *Wistar* rat	[73, 74]

	Methanolic extract		ATCC 43300 were 0.512 ± 0.03 mg/mL and 1024 ± 0.04 mg/mL. The methanolic extract increased VEGF and TGF-β1 genes expression in the HaCaT cells.	Topical application of ointment containing 10.0 or 20.0% of plant extract once a day/20 days after MRSA (ATCC 43300) infection.	
Nigella sativa Linn (Ranunculaceae)	Black seed<br><br>Oil	NI	The oil presented a dose dependent antibacterial activity against *S. aureus* isolated from wound of diabetic patients (including multidrug-resistant isolates) determined by the agar diffusion well assay	ND	[75]
Opuntia fícus-indica Miller (Cactaceae)	Flowers<br><br>Mucilage preparation from aqueous extract and methanolic extract	Glucose (32.4%) was the major monosaccharide in mucilage preparation.	Mucilage and methanolic extract presented antibacterial activity against *S. aureus*, determined by the agar diffusion well assay<br>Mucilage and methanolic extract exhibited antioxidant activity.	Excision wound in *Wistar* male rat. Topical treatment with 0.5 mg/mm^2 mucilage or methanolic extract was carried out once a day/ 13 days. Percentages of wound contraction of 80.0%, 90.0% and 98.0% were observed for the untreated, treated with Cicaflora cream® (reference drug), and treated with mucilage and methanolic extract animal groups, respectively; histological analysis indicated that the derma was properly arranged.	[76]
Parrotiopsis jacquemontiana (Hamamelidaceae)	Leaves<br><br>Oil	19 phytochemicals were identified, accounting 96.0% of the total oil. The major components were 2, 6-dimethyl-8-oxoocta-2, 6-dienoic acid, methyl ester (18.2%), syringol (17.8%), catechol (12.4%), guaiacol (5.2%), *p*-cresol (5.4%) and phenol, 2-propyl- (3.7%)	MIC and MBC values of the oil for *S. aureus* (including MRSA, and multidrug-resistant *S. aureus*) ranged from 32.0 to 128.0 μg/mL and 128.0 to 256.0 μg/mL, respectively.<br>The oil exhibited antioxidant activity.	Incisional wound model in Sprague-Dawley male rat<br>Cotton fabrics loaded with a 5.0 or 10.0% (w/v) oil solution in dimethylsulfoxide were applied to the wound and changed daily for up to 15 days. Percentages of wound contraction of 55.0%, 85.0% and 90.0%	[77]

				were observed for the untreated wound, treated with 5.0% and 10.0% oil, respectively. High rate of epithelialization, and increased hydroxyproline content (as indicator of collagen) were also observed during the healing process.	
Persea americana Mill. (Lauraceae)	Seeds<br><br>Methanolic extract	Alkaloids, anthocyanins, anthraquinones, flavonoids, polyphenols, saponins, steroids, tannins and triterpenes	MIC and MBC values were, respectively, 64.0 and 512.0 μg/mL for *S. aureus* ATCC 25923 and two clinical isolates.<br>At MIC value, the methanolic extract reduced the ATPases/H^+ proton pump activity and induced the proteins and reducing sugar leakage.	Excision wound in *Wistar* rat<br>Carbomer-based gels loaded with 1.0, 5.0 or 10.0% plant extract treatment (once a day/20 days) started 24 h after *S. aureus* infection.<br>Clindamycin (Aclin gel) and the ointment Baneocin®<br>(2%) 250 UI/5000 UI were used as reference drugs.	[78]
Piper betle L. (Piperaceae)	Leaves<br><br>Ethanolic extract	NI	Aqueous cream containing 2.5% or 5.0% of the extract inhibited the growth of *S. aureus* ATCC 25923 and one isolate of MRSA.	Excision wound in BALB/c male mouse<br>Aqueous cream containing 2.5% or 5.0% of plant extract application (once a day/14 days) started 24 h after *S. aureus* ATCC 25923 infection.<br>Mupirocin cream was used as reference drug.	[79]
Plukenetia volubilis L. (Euphorbiaceae)	Seeds<br><br>Oil	Polyunsaturated linoleic acid (35%) and linolenic acid (48%) were the major fatty acids identified.	The oil did not inhibit the growth of *S. aureus* CIP 53154, but was capable to inhibit the bacterial adhesion to keratinocytes.<br>The oil did not present citotoxicity to human keratinocyte cells or human skin explants.	ND	[80]

Portulaca oleracea	Trunk and leaves<br><br>Organic acids	α-eleostearic acid, palmitic acid, l-pyroglutamic acid, linoleic acid, stearidonic acid, azelaic acid, d-pantothenic acid, 6-hydroxypicolinic acid, and phloionolic acid correspond to 5.78% of the total organic acid content.	At MIC value (12.5 μg/mL) the organic acids caused significant morphological damage in planktonic cells of *S. aureus* (MRSA29, MRSA85 ATCC 29213), affecting the bacterial cell wall and membrane.	Incisional wound model in Kunming mouse<br>Oral treatment with organic acids – twice/day (125.0, 250.0 and 500.0 mg/kg/body weight) decreased the bacterial load (MRSA85) and inflammatory cytokines levels (IL-1β, IL-6, and TNF-α).	[81]
Quercus alba (Fagaceae)	Bark<br><br>Aqueous extract	NI	The aqueous extract at 100.0 and 150.0 mg/mL inhibited the growth of *S. aureus* ATCC 29213.	ND	[68]
Salvia kronenburgii Rech. f.<br>*Salvia euphratica* Montbret, Aucher & Rech. f. var. *euphratica* (Lamiaceae)	Aerial parts<br><br>Ethanolic extract	Total phenolic and flavonoid compounds were determined.	MIC value for both ethanolic extract was equal to 125.0 μg/mL for *S. aureus* ATCC 25925.<br>The extract presented antioxidant activity.	Excision and incisional wounds in diabetic Wistar albino male rats.<br>The ointment (containing 0.5% or 1.0% of the ethanolic extract) application was performed once a day/7 or 14 days.<br>The results were comparable with Fito®cream containing 15% (w/w) *Triticum vulgare* L. aqueous extract (reference drug) treatment.	[83]
Salvia officinalis L. (Lamiaceae)	Leaves<br><br>Essential oil	31 phytochemicals were identified, accounting 99.0% of the total oil. The major components were *cis*-thujone (26.8 %), camphor (16.4 %), *trans*-thujone (14.1 %) and 1,8-cineole (10.8 %)	MIC and MBC values were equal to 0.125 mg/mL for *S. aureus* ATCC 25923.	Excision wound in BALB/c male mouse<br>Ointment (containing 2.0 or 4.0% of essential oil) application (once a day/14 days) started after *S. aureus* (ATCC 25923) infection.<br>Mupirocin ointment was used as reference drug.	[84]

Salvia sclarea L. (Lamiaceae)	NI<br><br>Oil	The major component was linalool (12.4%), followed by α-pinene (4.5%), α-terpineol (3.5%), sabinense (3.3% and β-pinene (3.0%).	MIC ranged from 3.75 to 5.25 μL/mL for 27 *S. aureus* (11 MRSA and 16 MSSA) isolates.	ND	[85]
Sebastiania hispida (Mart.) Pax (Euphorbiaceae)	Leaves and stems<br><br>Ethanolic extract	Seven classes of secondary metabolites were identified. The major classes were phenolics, flavonoids, triterpenes and steroids.	The antibacterial activity of 0.2% and 2.0% extract concentration was evaluated by disk-diffusion assay against *S. aureus* ATCC 25923 and one MRSA clinical isolate. Only 2.0% extract inhibit the growth of both bacterial strains.	Excision wound in *Wistar* male rat Carbomer-based gel loaded with 0.2 or 2.0% of plant extract application (once a day/21 days) started after MRSA infection.<br>Kollagenase® ointment was used as reference drug	[86]
Syzygium aromaticum (Myrtaceae)	NI<br><br>Oil	Eugenol, caryophyllene, and 2-(octadecyloxy)-ethanol were the major compounds	The MIC and MBC values of clove oil for MRSA were 2.5 μL/mL and 5.0 μL/mL.<br>No antibacterial interaction between the plant oil and imipenem was observed.	Excision wound in *Wistar* rat Ointment (containing 5.0 or 10.0% of clove oil) treatment (once a day) started after MRSA infection.<br>Imipenem was used as reference drug	[87]
Urtica dioica (Urticaceae)	Leaves<br><br>Hydroethanolic extract<br>Ethyl acetate fraction	Alkaloids, tannins, polyphenols, and flavonoids. The main identified polyunsaturated fatty acids were palmitoleic, oleic, linoleic, and linolenic acids. Palmitic acid was the most common saturated fatty acid. Lupeol, a triterpene, accounted for about 86% of the total sterols.	MIC = 5.0 mg/mL of the hydroethanolic extract and ethyl acetate fraction for *S. aureus* ATCC 25923.<br>The extract presented antioxidant activity.	Excision wound model in *Wistar* rat. Ointment (containing 10% of plant extract) application (every two days/11 days) induced wound contraction around 92.0%.<br>The results were comparable with CICAFLORA cream (reference drug) treatment that induced around 85.0% wound contraction after 11 days.<br>Moreover, the plant extract ointment induced significant neovascularization and re-ephitelialization at the wound site.	[88]
Zanthoxylum nitidum (Roxb.) DC.	Dried roots	Isoquinoline alkaloids:	MIC values ranged from 8.0 to 32 μg/mL for 6-ADHF; and 8.0 to 64.0	Incisional wound model in Kunming male mouse.	[89]

(Rutaceae)	NI	6-acetonyl-dihydrofagaridine (6-ADHF)<br>6-acetonyl-dihydrochelerythrine (6-ADHC)	μg/mL for 6-ADHC for *S. aureus* ATCC 25923 and two MRSA isolates, and both compounds exhibited a synergistic interaction with ampicillin against MRSA, inhibiting the growth of planktonic cells and erradicating biofilm cells.	Treatment (twice a day/3 days) with the compounds combined or not with ampicillin started one day after MRSA inoculation.	

MIC: minimal inhibitory concentration; MBC: minimal bactericidal concentration; CC_{50}: Cytotoxic concentrations of the extract/compound capable of reducing mammalian cells viability in 50%; ATCC: American Type Culture Collection; ND: not determined; NI: not identified; MRSA: methicillin-resistant *Staphylococcus aureus*. Ref.: Reference. HaCaT: human adult skin keratinocytes; HL60: human promyelocytic leukemia cells; NIH3T3 embryonic mouse fibroblast cells; TNF: Tumor Necrosis Factor, NO: nitric oxide; VEGF: Vascular Endothelial Growth Factor; TGF: Transforming Growth Factor.
